# Posterior Capsular Opacification and Glistening in Hydrophobic Monofocal Biaspheric Intraocular Lens Two Years After Implantation: A Case Control Study

**DOI:** 10.1155/joph/3520219

**Published:** 2024-12-31

**Authors:** Ana Hervás-Ontiveros, Enrique España-Gregori, Carlos Fresno-Cañada, Rodrigo Butrón-Ruíz, Alejandro Cerviño

**Affiliations:** ^1^Department of Ophthalmology, Hospital Universitario La Fe, Valencia, Spain; ^2^Department of Ophthalmology, Hospital 9 de Octubre, Valencia, Spain; ^3^Department of Ophthalmology, Hospital Comarcal de Vinaroz, Vinaroz, Spain; ^4^Department of Optics & Optometry & Vision Sciences, University of Valencia, Valencia, Spain

**Keywords:** efficacy, glistening, posterior capsule opacification, safety

## Abstract

**Background:** This study aims to analyze the prevalence and severity of posterior capsule opacification (PCO) and glistening in a new hydrophobic biaspheric monofocal intraocular lens (IOLs) 24 months after implantation.

**Methods:** By means of a ambispective, observational, case-control design, a total of 297 eyes from 200 cataract surgery patients were included in the study (118 females and 82 males; mean age: 72.31 ± 9.87 years, ranging from 35 to 92) and examined at the Hospital Universitario y Politécnico la Fe, Valencia (Spain). Data corresponding to patients implanted with either Asqelio (Study IOL) or Clareon (Control IOL) monofocal IOLs at least 24 months prior to study visit were analyzed. Prevalence and intensity of PCO and IOL glistening were determined and graded for both groups by a single masked observer. Refractive outcomes by autorefractometry, visual acuity, and wavefront aberrations determined by ray tracing were also measured and compared.

**Results:** Prevalence of PCO in patients implanted with the Study IOL 24 months after implantation was 4.0%, lower than that for the Control IOL. Intensity of PCO in both groups was low. One lens in each group presented a Grade 1 glistening after 24 months from implantation. Differences in visual acuity between Study and Control Groups were not significant (*p*=0.260 and 0.115 for UDVA and CVA, respectively). Residual spherical aberration was significantly lower in the Study Group than that in the Control Group (*p*=0.007).

**Conclusion:** Prevalence of PCO was considerably lower for Asqelio IOL than for the Control IOL and reports available in the literature for other hydrophobic IOLs. Prevalence of glistening was minimal in both study and control IOLs.

**Trial Registration:** ClinicalTrials.gov identifier: NCT04971863

## 1. Introduction

Intraocular lens (IOLs) implantation has revolutionized cataract surgery, providing patients with restored vision and enhanced quality of life. However, despite the significant advancements in surgical techniques and materials, certain complications such as posterior capsular opacification (PCO) and material glistening continue to challenge ophthalmic surgeons and compromise visual outcomes.

PCO, characterized by the proliferation and transformation of residual lens epithelial cells leading to fibrotic changes and pearl formation on the posterior capsule, remains the most common long-term complication following cataract surgery, impacting visual acuity and often necessitating additional interventions [1]. Nd:YAG laser-assisted posterior capsulotomy presents potential risks [2, 3] and, therefore, PCO prevention is of most clinical interest.

Similarly, the appearance of microvacuoles within the IOL material raises concerns regarding optical quality and long-term stability since the increased light scattering produced by these microvacuoles [4], known as glistening, deteriorates the optical quality of the IOL [5]. Glistening formation is particularly noted in certain hydrophobic acrylic IOLs, especially those that are injection molded, due to microstructural changes in the polymer matrix resulting from manufacturing processes.

The choice of IOL material and design plays a crucial role in determining the prevalence and severity of these complications [6]. Material properties such as biocompatibility, surface characteristics, and resistance to environmental factors profoundly influence the long-term performance and safety of IOLs [7].

Hydrophobic acrylic IOL are more widely used on the market nowadays [8], as they can have thinner profiles with higher refractive index and adhere tightly to the capsule, reducing epithelial cell proliferation [9] and, therefore, reducing the incidence of PCO. Moreover, a sharp-edged design is easier to apply to hydrophobic IOLs, preventing migration of lens epithelial cells to the IOL [10]. Glistening is also found to be more likely to occur in hydrophobic IOL [11] because water collection in hydrophobic polymers, forming vacuoles that expand with temperature fluctuations.

Thus, understanding the underlying mechanisms contributing to PCO and material glistening is imperative for optimizing patient outcomes and ensuring the longevity of visual rehabilitation.

The purpose of this study is to assess the prevalence and intensity of PCO and glistening at least 24 months after surgery in patients implanted with Asqelio IOL (AST VisionCare, Inc., Billerica, MA, USA). The Asqelio IOL is made of a relatively new hydrophobic, very low water content, high Abbe number, and soft material, and lenses are manufactured by cryo-lathing. In order to verify these parameters and to evaluate the material stability during this period, a control group of a well-known and widely studied hydrophobic IOL model was included.

## 2. Methods

This observational, ambispective (retrospective/prospective), case-control, postmarketing study of CE-marked medical devices was carried out at the Hospital Universitario y Politécnico la Fe, in Valencia (Spain). The study was approved by the Ethics Committee of the Hospital Universitario y Politécnico la Fe (Valencia, Spain) and was conducted in accordance with the tenets of the Declaration of Helsinki. Written informed consent was obtained from all patients prior to their enrollment in this study and possible consequences of the study were explained.

### 2.1. Patients

Inclusion criteria included patients 22 years of age or older who had undergone cataract surgery on at least one eye and implanted with one of 2 lenses: Asqelio monofocal IOL (Study Group) or Clareon monofocal IOL (Control Group). Postoperative emmetropia was planned (±0.5D spherical equivalent) for all patients. Exclusion criteria included the following: any pathology that reduces potential visual acuity with its best correction beyond 0.30 logMAR (including amblyopia, severe corneal dystrophy, diabetic retinopathy, extremely narrow anterior chamber depth, microphthalmos, retinal detachment, corneal transplantation, severe recurrent anterior or posterior segment inflammation of unknown etiology, iris neovascularization, uncontrolled glaucoma, clinically significant macular degeneration, or diagnosis of pseudoexfoliation); previous corneal surgery or trauma; rubella or surgery caused by traumatic cataract; eye trauma or refractive surgery; and any other ocular or systemic condition that, in the opinion of the investigator, should exclude the patient from the study. Those patients who require additional procedures due to intraoperative complications, mechanical or surgical interventions to manipulate the pupil, excessive iris mobility, significant vitreous loss, significant anterior chamber hyphema, zonular capsular rupture, or other unrecognized ocular conditions that could compromise the positioning of the IOL, as well as the impossibility of placing the IOL in the capsular pouch due to surgical complications, will be excluded from the study.

### 2.2. IOLs

The Study lens was the Asqelio monofocal IOL (model QLIO130C; AST VisionCare, Inc., Billerica, MA, USA). This IOL has a biaspheric geometry, with a total diameter of 13.0 mm, C-Loop platform, and 360 degrees sharp edge. It is made of soft hydrophobic acrylic material (glistening free) with a refractive index of 1.50, UV absorber, Abbe number of 50, water content < 0.5%, and a spherical aberration of −0.27 microns.

The Control lens was the Clareon monofocal IOL (model SY60CL; Alcon Vision LLC, Fort Worth, TX, USA). It is made of one of the most modern IOL materials, approved by the FDA in January 7^th^ of 2020, and with relevant studies, both clinical [12] and laboratory settings [13], validating its stability against the appearance of PCO, as well as the efficacy and safety of its implantation. For this reason, in the present study, the Clareon IOL was used as control.

Only clear lenses were implanted in patients from both groups.

### 2.3. Variables

#### 2.3.1. Visual Acuity

Monocular logMAR distance visual acuity with (CDVA) and without correction (UDVA) under photopic conditions were determined at a distance of 4 m using the Early Treatment Diabetic Retinopathy Study (ETDRS) letters optotype. The percentage of patients with monocular CDVA of 0.3 logMAR or better 24 months after surgery was compared with the historical safety and efficacy rate referred to in EN ISO 11979-7:2014. A higher percentage would indicate good visual outcomes, with most patients achieving visual acuity of 0.3 logMAR or better.

#### 2.3.2. Refraction

Refraction was determined using automated objective refractometer.

#### 2.3.3. Prevalence and Severity of PCO

Evaluation of PCO was performed by slit lamp observation using the gradation method developed by Tetz et al. [14]. Images were subjectively graded: 0 (absent): clear capsule; 1 (minimal): slight wrinkles, sheets of epithelial cells; 2 (mild): honeycomb PCO patterns, thicker homogeneous layers, denser fibrosis; 3 (moderate): classic Elschnigg pearls, and very thick homogeneous layers; and 4 (severe): very thick Elschnigg pearls, with a darkening effect or any kind of severe opacification. A single investigator, masked to the visual results and the type of IOL implanted, performed all PCO gradations after the examination of the patient by the other investigators, based on the images taken by backlighting with the dilated pupil. Prevalence and severity were compared between both groups.

#### 2.3.4. Prevalence and Severity of IOL Glistening

The prevalence and severity of glistening (inclusions in IOL material that generate light scattering) were assessed by examining the center of the IOL optical zone with a dilated pupil using a 10 × 2 mm [2] slit lamp beam at a 30-degree angle. The intensity of glistening was graded according to the classification proposed by Tognetto [15] as the number of glistening in the central region according to the following scale: 0 = absent, 1 = traceable (countable vacuoles), 2 = moderate (low density of uncountable vacuoles), and 3 = severe (high density of uncountable vacuoles). A single investigator, masked to the visual results and the type of IOL implanted, performed all glistening gradations after the examination of the patient by the other investigators, based on the images taken. Prevalence and severity were compared between both groups.

#### 2.3.5. Ocular Optical Quality

The iTrace ocular aberrometry system (Tracey Technologies, Houston, TX, USA) was used. This system allows the determination of aberrations of the eye's wavefront using ray-tracing technology, as well as corneal topography using a Placido disc, which allows the contribution of the cornea to the optical quality of the eye to be calculated. Total wavefront aberrations root-mean-square (RMS), spherical aberration, and coma coefficients were obtained for a 6 mm pupil size and compared between both groups.

#### 2.3.6. Adverse Events (Ocular and Nonocular and Serious and Nonserious), Including Secondary Surgical Interventions

Adverse events were recorded from solicited and spontaneous comments from patients and from observations made by the investigator.

### 2.4. Sample Size

An investigator was in charge of selecting the patients in the databases that a priori meets the inclusion criteria to participate in the study and cited them to carry out the basic postoperative follow-up examination, checked that they did not present any exclusion criteria, and, where appropriate, explained the nature of the study and obtain informed consent. This researcher was the only one to know the type of IOL implanted in each of the patients. Both the principal investigator and the rest of the researchers in charge of taking the measurements of the parameters under study, as well as carrying out the assessments of the PCO and glistening images, were not aware of type of IOL was implanted in each patient. The calculation of the minimum sample size was carried out using the Power G program, considering the reference values obtained in the literature for different IOLs in the market [16], as well as the prevalence values of PCO in patients implanted with the Clareon IOL taken as a reference [17]. The calculations were performed using the nominal test of proportions (Chi-square) for a statistical power of 0.8 and an alpha of 0.05, with the result being 116 eyes for each type of IOL.

### 2.5. Data Analysis

Data analysis was performed using IBM SPSS Statistics v28.0 (IBM Corp, Armonk, NY, USA) and Microsoft Excel for Mac v16.41 (Microsoft Co, Redmond, WA, USA). The significance level was set at *p* < 0.05. Categorical variables were described in the form of frequencies and percentages and continuous variables in means and standard deviations. Histograms of postoperative refractive error and refractive cylinder were built to assess refractive accuracy. Cumulative histogram of postoperative visual performance was built to assess efficacy of refractive correction. The normality of data distribution as determined by the Shapiro–Wilk test and, based on this distribution, the unpaired *t*-test or Mann–Whitney–Wilcoxon test was used for comparisons between groups when appropriate.

Preoperative clinical data were retrospectively collected from the medical history of the patient. Therefore, data analysis that comprised this data was limited to the number of patients for whom this information was available. Missing data were excluded from analysis.

## 3. Results

A total of 296 eyes from 200 cataract surgery patients were included in the present analysis (118 females and 82 males; mean age: 72.31 ± 9.87 years of age ranging from 35 to 92). Initially, a total of 150 subjects implanted with the Asqelio™ IOL and another 150 implanted with the monofocal Clareon™ IOL who had undergone cataract surgery and IOL implantation at least 24 months prior to the study visit were identified from hospital records and initially aimed as study sample. The database was closed after reaching 100 patients per group since further patient enrollment in the study group became unfeasible due to several factors (e.g., presence of other ocular pathologies, poor cooperation, lost to follow-up, or patient fatalities).

In accordance with the ambispective design and the inclusion criteria specified, patients would be examined at 24 months postsurgery. However, due to the retrospective nature of patient recruitment, differences in surgical dates, and scheduling constraints for the study visit, the actual timing of follow-up assessments varied. As a result, while all patients met the minimum follow-up requirement, some were seen slightly before or after the ideal 24-month mark. This led to mean postoperative intervals of approximately 23 ± 44 months for the study group and 25 ± 10 months for the Control Group.

Table 1 displays the preoperative data of the eyes included in the analysis, grouped by IOL implanted. The table also includes the significance level after the Mann–Whitney *U* test for unpaired samples. Bold *p* values highlight those for which there was a significant difference between both groups.

### 3.1. Postoperative Refraction

**Figure 1:**
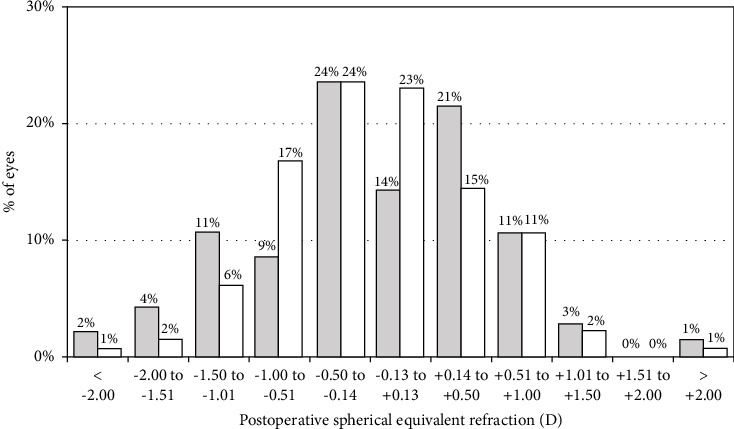
Spherical equivalent refractive accuracy for the Study Group (gray bars) and Control Group (white bars).

The average spherical equivalent was −0.20 ± 0.93D at 24-month postop for eyes in the Study Group and −0.14 ± 0.78D for eyes in the Control Group. Differences were not statistically significant (*p*=0.273).  Figure 1 shows the distribution of spherical equivalent for both groups.

**Figure 2:**
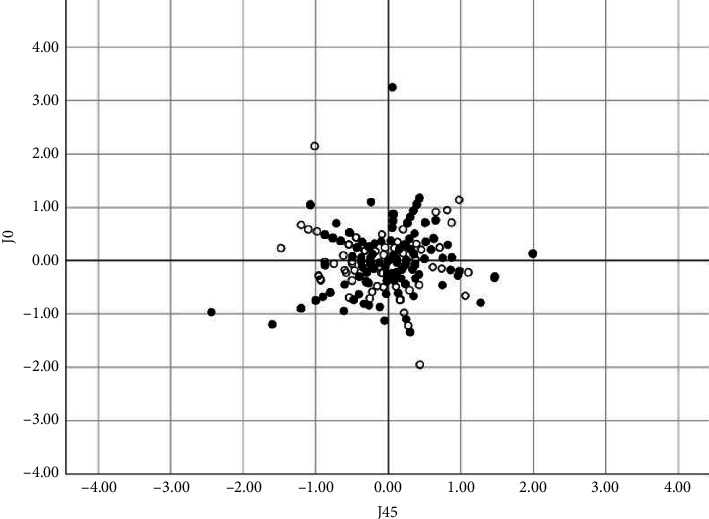
Scatterplot representing the distribution of residual astigmatism (D) scatterplot (J_0_ vs. J_45_) for the Study Group (black bins) and Control Group (empty bins).

The average residual cylinder was −1.33 ± 0.99 D for the Study Group and −1.29 ± 0.86D for the Control Group. Differences were not statistically significant between the cylindrical vector components in both groups (*p*=0.377 and 0.422 for J_0_ and J_45_, respectively).  Figure 2 shows the scatterplots for the astigmatism components (J_0_ and J_45_) in both groups.

### 3.2. Prevalence and Intensity of PCO

**Figure 3:**
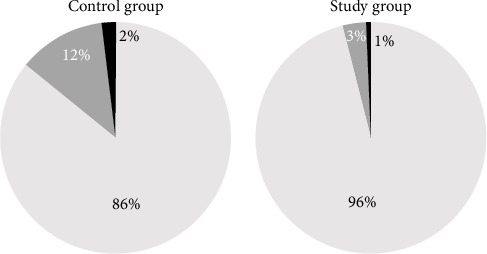
Pie charts displaying the prevalence of PCO gradings in the Control Group sample (left, *n* = 146 eyes) and Study Group sample (right, *n* = 151 eyes). White: no PCO; gray: PCO Grade 1; black: PCO Grade 2.

In the overall sample analyzed in the present report (*n* = 297 eyes), only 27 eyes were reported as having some degree of PCO at 24-month postop. These were graded according to Tetz et al. [14] and 23 were found a Grade 1 (minimal PCO: slight wrinkles and sheets of epithelial cells) and 4 as Grade 2 (mild PCO: honeycomb PCO patterns, thicker homogeneous layers, and denser fibrosis). There were no cases of higher degrees of PCO in either group. Out of these cases, 18 eyes within the Control Group were graded as having Grade 1 PCO and 3 as Grade 2. Within the Study Group sample, 5 eyes were graded as Grade 1 and 1 as Grade 2 (Figure 3).

Average PCO scores were 0.05 ± 0.24 and 0.16 ± 0.42 in Study and Control Groups, respectively, being the differences statistically significant (*p*=0.002). In addition, six eyes in the Control Group (4%) and one in the Study Group (0.6%) had had Nd:YAG capsulotomy prior to the study visit.

### 3.3. Prevalence and Intensity of Glistening

Glistening was found in 1 lens from each group at the 24-month visit. In both cases, it was scored as grade 1 according to the classification proposed by Tognetto [15], i.e., traceable (countable vacuoles).

### 3.4. Visual Performance

Table 2 displays the average visual acuities obtained for distance vision at 24-month postsurgery.

### 3.5. Ocular Optical Quality

The average RMS postop was 0.54 ± 0.22 microns for the Control Group and 0.57 ± 0.22 microns for the Study Group samples. Differences were not statistically significant (*p*=0.196). The mean spherical aberration values were 0.02 ± 0.15 microns for the Control Group (*n* = 141) and −0.01 ± 0.01 microns for Study Group, being significantly different between both groups (*p*=0.007).

### 3.6. Adverse Events

Other than the aforementioned PCO cases, two eyes in the Study Group, belonging to the same patient, showed cornea verticillata, and one eye in the Control Group showed pterygium. In both cases, these adverse events were unlikely related to the IOL.

## 4. Discussion

In the present study, the main outcome measure was the safety of implantation of Asqelio monofocal IOL after cataract surgery, using Clareon monofocal IOL as control IOL. Safety was assessed by monitoring adverse events, including secondary surgical interventions, with particular emphasis in assessing the prevalence and intensity of PCO and IOL glistening. Prevalence of PCO is influenced by patient-related, surgery-related, and IOL-related factors. The IOL-related factors associated with PCO are an edge-shaped and the surgical technique [18], but also optics edge geometry and haptics shape and angulation play a role. All the lenses included in the present study, in both Study and Control Groups, were implanted by the same experienced surgeon (AHO). A meta-analysis of randomized controlled trials reported lower PCO rates for hydrophobic versus hydrophilic IOLs at 1- and 2-years follow-up, as well as significantly lower rates of Nd:YAG capsulotomy [19]. In a study by Lehmann et al., the rate of PCO reported for the Clareon IOL was 5.4% (19/350), and the rate of Nd:YAG capsulotomy was 4.6% (16/350) 12 months after the surgery [12]. A comparable performance has been found in the ex vivo formation of PCO between Clareon and AcrySof (Alcon Vision LLC, Fort Worth, TX, USA) IOLs [20]. In a recent clinical study assessing AcrySof IOLs, PCO developed in 11.6% of the 164 included eyes at 6-month postop, being not clinically significant [21]. In the analysis included in the present study, the rate of PCO for the Control Group was considerably higher than those reported in the literature, 14.4% (21/146), but it must be noted that most of those cases were graded at the mildest level. In any case, the outcomes of this analysis allow confirming the prevalence of PCO in eyes implanted with Asqelio IOLs 24 months after implantation (4.0%) as being low, both in prevalence and intensity, not worse than control IOLs, and lower than available reports with other IOLs.

Glistenings are linked to IOL characteristics, such as material, manufacturing process, and temperature fluctuations, and are more common in hydrophobic IOLs [22–24]. Newer materials, such as those used in both IOLs included in the present study, have been developed to minimize glistening formation. In an in vitro study comparing different commercially available IOLs, the Clareon IOL was found as having the lowest levels of glistenings [13]. Lehmann et al. [12] did not find any glistenings in Clareon IOLs in a 12-month follow-up study. These outcomes agree with the findings of the present analysis, with only one case of glistening in the Clareon IOL group, and one in the Asqelio IOL group, showing that the prevalence and intensity of glistening after 24-month postop was the same in both Study and Control Groups.

Efficacy is determined as the percentage of eyes with CDVA of 0.3 LogMAR or better. Lehmann et al. [12] reported that in their study at month 12 after implantation, 99.7% of patients achieved CDVA of 0.3 logMAR or better, and the one-sided 95% upper confidence limit of 99.99% was greater than the SPE rate of 92.5% [25]. The outcomes of the present study confirm that the visual performance with Asqelio monofocal IOL is not worse than that Clareon monofocal IOL.

Asqelio IOLs have a biaspheric design with a spherical aberration of −0.27 microns for a 6 mm pupil size. The biasphericity allows more degrees of freedom in IOL design to achieve this amount of induced spherical aberration that would allow compensating the corneal spherical aberration present in most of the population and that according to different studies is around 0.27 ± 0.02 [26] or 0.28 ± 0.09 [27] microns. The outcomes of the present study do show Asqelio IOLs as significantly more efficient in compensating corneal spherical aberration than Clareon IOLs (*p*=0.007), although the mean difference of 0.04 ± 0.02 microns found in the present sample is unlikely to yield any clinical implications.

Note that this study has several limitations. It has an ambispective design, meaning that patients enrolled were implanted prior to enrollment, and, therefore, there is a lack of control on measurement acquisition prior to the study visit as well as missing preoperative data for analysis. Another limitation of the present analysis is the inclusion of both eyes of those patients bilaterally implanted. Since the variance between eyes is usually less than that between patients, the overall variance of a sample of measurements combined from both eyes could be an underestimate of the true variance resulting in an increased risk of a Type 1 error.

To conclude, the findings from this study support the safety of Asqelio monofocal IOL for refractive correction following cataract extraction. Notably, the clinical performance of the Asqelio IOLs was comparable with that of control lenses. The stability and transparency of the material persist after 24 months postimplantation, indicating its enduring quality. Importantly, the prevalence and severity of PCO were significantly reduced in comparison with control lenses. This reduced prevalence aligns with lower rates than those reported in the literature for other IOLs.

## Figures and Tables

**Table 1:** Preoperative descriptive statistics of the sample.

	Study Group			Control Group			*p* value
	*n*	Mean	SD	*n*	Mean	SD	
Preop SE (D)	79	0.21	2.84	37	−0.48	4.27	0.187
Preop J0 (D)	79	0.04	0.64	37	0.08	0.68	0.380
Preop J45 (D)	79	−0.04	0.60	37	0.08	0.54	0.131
IOP (mmHg)	91	16.63	2.89	69	15.51	2.81	**0.007**
AL (mm)	135	23.27	0.98	141	23.83	1.54	**< 0.001**
ACD (mm)	133	3.11	0.49	141	3.26	0.44	**0.005**
K1 (D)	134	43.32	1.70	141	42.97	2.10	0.062
K2 (D)	134	44.39	1.83	141	44.25	2.03	0.269
WTW (mm)	61	11.56	0.81	72	11.72	0.70	0.111
IOL power (D)	145	21.99	2.85	139	20.80	4.03	**0.002**

*Note:* Bold *p* values indicate statistical significance for the differences between groups. K1: flat corneal meridian; K2: steep corneal meridian.

Abbreviations: ACD, anterior chamber depth; AL, axial length; IOL, intraocular lens; IOP, intraocular pressure; SD, standard deviation; SE, spherical equivalent; WTW, white-to-white distance.

**Table 2:** Monocular visual acuity obtained for distance 24 months after surgery.

	Study Group		Control Group		*p* value
	Mean	SD	Mean	SD	
UDVA	0.16	0.17	0.14	0.18	0.260
CDVA	0.12	0.18	0.09	0.09	0.115

*Note:* All values are in LogMAR units.

Abbreviations: CDVA, best-corrected distance visual acuity; SD, standard deviation; UDVA, uncorrected distance visual acuity.

## Data Availability

The data that support the findings of this study are available from the corresponding author upon reasonable request and with permission of AST VisionCare, Inc..

## Nomenclature

IOL: Intraocular lens
PCO: Posterior capsule opacification
CDVA: Corrected distance visual acuity
UDVA: Unicorrected distance visual acuity
ETDRS: Early treatment diabetic retinopathy study
RMS: Root-mean-square
